# Neuroprotective Effects of Daphnetin against NMDA Receptor-Mediated Excitotoxicity

**DOI:** 10.3390/molecules190914542

**Published:** 2014-09-15

**Authors:** Le Yang, Qi Yang, Kun Zhang, Yu-Jiao Li, Yu-Mei Wu, Shui-Bing Liu, Lian-He Zheng, Ming-Gao Zhao

**Affiliations:** 1 Department of Pharmacology, School of Pharmacy, Fourth Military Medical University, Xi’an 710032, China; E-Mails: yanglefmmu@163.com (L.Y.); yangqifmmu@126.com (Q.Y.); kunzhang1900@163.com (K.Z.); yujiao_li@126.com (Y.-J.L.); yumeiwu@fmmu.edu.cn (Y.-M.W.); liushb1974@aliyun.com (S.-B.L.); 2 Department Department of Orthopaedics, Tangdu Hospital, Fourth Military Medical University, Xi’an 710032, China

**Keywords:** daphnetin, excitotoxicity, neuron, apoptosis, calcium

## Abstract

The accumulation of glutamate can excessively activate the *N*-methyl-d-aspartate (NMDA) receptors and cause excitotoxicity. Daphnetin (Dap), a coumarin derivative, is a protein kinase inhibitor that exhibits antioxidant and neuroprotective properties. However, little is known about the neuroprotective effects of Dap on glutamate-induced excitotoxicity. We evaluated the neuroprotective activities in the primary cultured cortical neurons against NMDA-induced excitotoxicity. Pretreatment with Dap significantly prevented NMDA-induced neuronal cell loss. Dap significantly inhibited the neuronal apoptosis by regulating balance of Bcl-2 and Bax expression. Furthermore, pretreatment of Dap reversed the up-regulation of NR2B-containing NMDA receptors and inhibited the intracellular Ca^2+^ overload induced by NMDA exposure. In addition, Dap prevented cerebral ischemic injury in mice induced via a 2 h middle cerebral artery occlusion and a 24 h reperfusion* in vivo*. The findings suggest that Dap prevents the excitotoxicity through inhibiting the NR2B-containing NMDA receptors and the subsequent calcium overload in cultured cortical neurons.

## 1. Introduction

**Figure 1:**
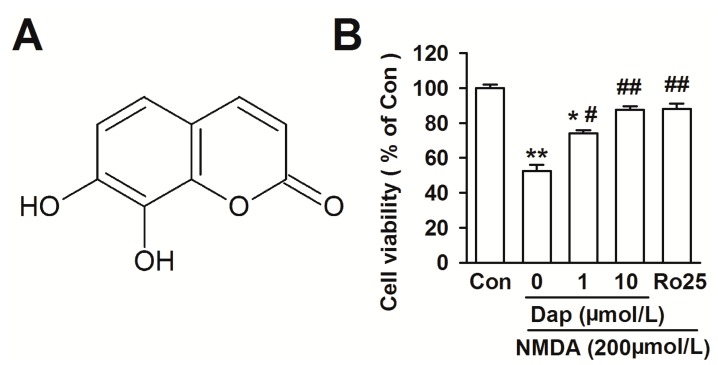
Effects of Dap on cell viability in cultured cortical neurons. (**A**) Chemical structure of Dapexin. (**B**) Effects of Dap (0.1, 1 and 10 μM) and Ro 25-6981 (0.3 μM) on the cell viability of cortical neurons after exposure to NMDA. The data were pooled from three independent experiments. * *p* < 0.05, ** *p* < 0.01 compared with control. ^#^ *p* < 0.05, ^##^ *p* < 0.01 compared with NMDA alone.

Daphnetin (7,8-dihydroxycoumarin, Dap, Figure 1A) extracted from *Daphne odora Var. marginata* (*D. marginata*) mainly contains coumarin compounds. Among these coumarins, daphnetin has been reported to make important contributions to the analgesic and anti-inflammatory activity of *Daphne odora* [1]. It has been clinically used in the treatment of coagulation disorders and rheumatoid arthritis [2]. Recently, it is reported that Dap significantly increases neurite outgrowth and promotes neuronal survival in primary cultured rat cortical neurons. The neurotrophic effects of Dap are probably associated with increased BDNF expression [3].

Glutamate is a primary excitatory neurotransmitter and plays a key role in synaptic plasticity, learning and memory and excitotoxicity [4]. Glutamate accumulation can cause *N*-methyl-d-aspartate (NMDA) receptor-mediated excitotoxicity, which has been implicated in neurodegeneration [5]. NMDA receptors, a kind of glutamate-gated ion channels, are widely expressed in the central nervous system and highly permeable to calcium ions [6]. The overactivation of glutamate receptors can trigger high calcium (Ca^2+^) influx, which activates a number of enzymes that damage cell structures. This Ca^2+^ influx is thought to contribute to Ca^2+^-mediated excitotoxic neuronal cell death in the above-mentioned disease processes [6]. In mammalian central neurons tyrosine phosphorylation regulates the function of the NMDA receptor and protein-tyrosine phosphorylation potentiates the NMDA currents which are important in neuronal development, plasticity and toxicity [7]. Dap strongly inhibits not only epidermal growth factor (EGF) receptor catalyzed tyrosine phosphorylation of exogenous substrate but also PKA and PKC activities. The half maximal effect (IC50) of daphnetin is at 7.7 μM for EGF receptor tyrosine kinase, 9.3 μM for PKA and 25.0 μM for PKC [8]. Recent study reported neuroprotection of Dap against glutamate-induced toxicity in HT22 cells and ischemic brain injury [9]. However, the underlying mechanisms of neuroprotection of Dap against excitotoxicity is not well known. The aim of this study was to investigate the possible protective efficacy of Dap in neuronal apoptosis induced by NMDA receptor activation and to elucidate the underlying mechanisms. We found that pretreatment of Dap significantly attenuated exitotoxicity by depressing the apoptotic signaling pathways.

## 2. Results and Discussion

### 2.1. Effects of Dap on Cell Viability of Cortical Neurons

NMDA receptor has been proven to be involved in the pathogenesis of neurodegenerative disorders associated with glutamate excitotoxicity. The neurons exposed to NMDA (200 μM) for 30 min induced significant cell injury as shown by the cell viability measured by the MTT assay (cell viability in NMDA treatment: 52.3% ± 3.8%, *p* < 0.01* versus* control; Figure 1B). Neurons were pretreated with Dap (0.1, 1, 10 μM) for 24 h, and then treated with NMDA (200 μM) and glycine (10 μM) for another 30 min. The cells were returned to the original culture medium containing Dap 24 h. Pretreatment with Dap significantly prevented the cell loss induced by the NMDA exposure. Neuroprotection of 10 μM Dap was most effective (87.5% ± 2.1%, *p* < 0.01 compared with NMDA alone). Dap (0.1, 1 and 10 μM) alone showed no effect on cell viability (data not shown). In addition, NR2B antagonist Ro25-6981 (0.3 μM) was added in the medium as same time as NMDA exposure. It blocked cell death caused by NMDA exposure, suggesting that the NR2B-containing NMDA receptor subtypes mediate the excitotoxicity.

### 2.2. Anti-Apoptotic Activities of Dap

**Figure 2:**
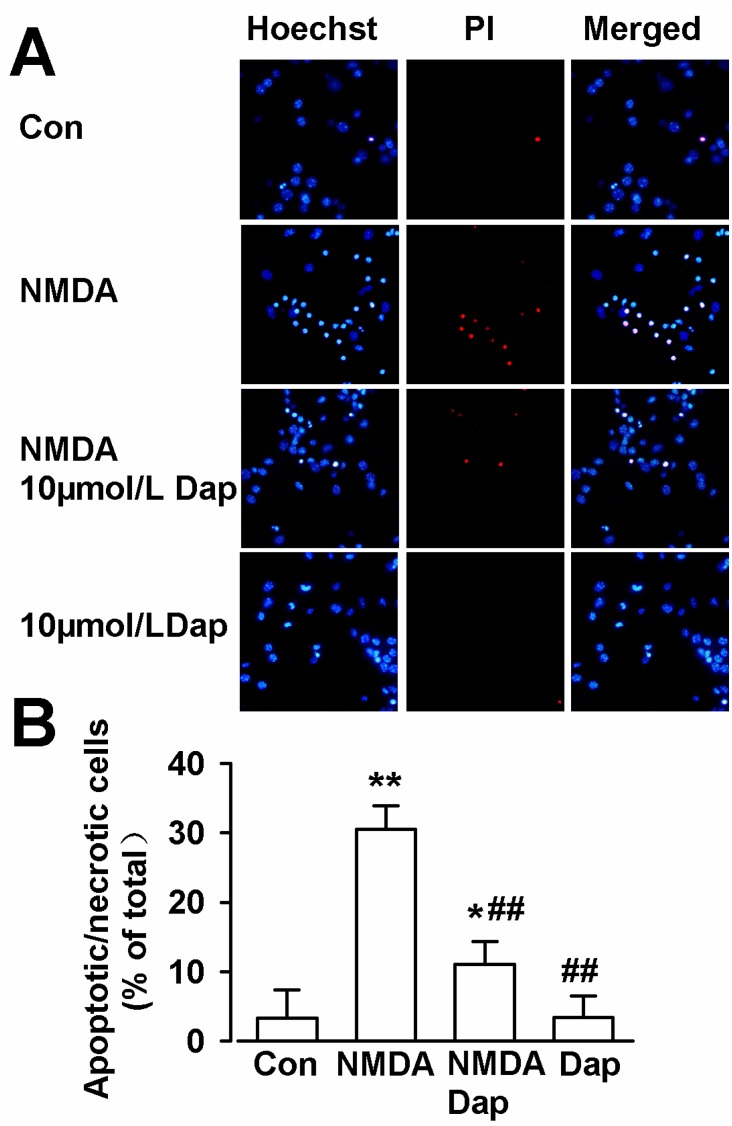
Hoechst 33258 and PI double staining in cultured cortical neurons. (**A**) The representative fluorescence images obtained after Hoechst 33258, PI, and double staining in cortical neurons. Scale bar: 20 μm. (**B**) The percentage of apoptotic neurons in total neurons for control, NMDA, NMDA + Dap (10 μM), and Dap (10 μM) treated groups. The apoptotic numbers were counted from three independent experiments. * *p* < 0.05, ** *p* < 0.01 compared with control, ^##^ *p* < 0.01 compared with NMDA alone.

Hoechst 33258 can penetrate the cell membrane of necrotic and living cells, but PI can not pass through the living cells. Therefore, PI is usually used to detect cell apoptosis and necrosis. Next, hoechst 33258 and PI double-staining were performed to further determine the neuroprotective effects of Dap on NMDA-induced apoptosis in cultured cortical neurons. In the normal conditions, the cells underwent less cell death as compared to the cells in the NMDA injury group (control: 3.3% ± 4.1%; NMDA: 30.6% ± 3.3%; *p* < 0.01 compared with control; Figure 2A,B). Pretreatment with Dap (10 μM) significantly decreased the number of apoptotic and necrotic cells (11.1% ± 3.3%, *p* < 0.01; compared with NMDA alone; Figure 2A,B). Treatment of Dap (10 μM) alone showed no effect on apoptosis or necrosis. These data suggest that Dap protects neurons from apoptosis or necrosis mediated by NMDA.

### 2.3. Effects of Dap on the Expression of Apoptotic Proteins

**Figure 3:**
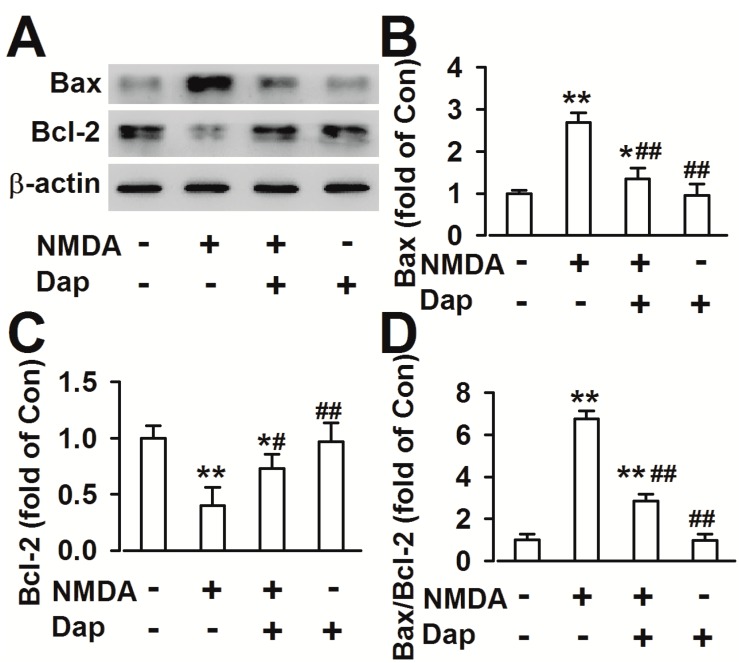
Effects of Dap on the levels of Bax and Bcl-2 (**A**) Expression levels of Bax and Bcl-2 proteins were detected by Western blot. (**B**) The result showed that the levels of Bax markedly increased after exposure to NMDA in cultured cortical neurons. Pretreatment of 10 μM significantly decreased the levels of Bax. (**C**) The levels of Bcl-2 significantly decreased after exposure to NMDA in cultured cortical neurons. Pretreatment of 10 μM Dap evidently increased the levels of Bcl-2. (**D**) The ratio of Bax/Bcl-2 was notably increased after NMDA exposure; however, the 10 μM Dap reversed the ratio. The data were pooled from five independent experiments. * *p* < 0.05, ** *p* < 0.01 compared with control, ^#^ *p* < 0.05, ^##^ *p* < 0.01 compared with NMDA alone.

Bcl-2 family members include both anti-apoptotic (e.g*.*, Bcl-2 and Bcl-xl) and pro-apoptotic (e.g., Bax, Bad, Bak, and Bid) proteins. The ratio between the two subsets determines the susceptibility of the cells to a death signal [10]. Dap (10 μM) treatment alone did not alter the expression of these proteins (Bcl-2: 0.95 ± 0.27; Bax: 0.91 ± 0.17 fold of the control). Compared with control, 200 μM NMDA exposure in cultured cortical neurons increased the levels of Bax (2.68 ± 0.24 fold of the control, *p* < 0.01 compared with control; Figure 3A,B), decreased the levels of Bcl-2 (0.40 ± 0.16 fold of the control, *p* < 0.01 compared with control; Figure 3A,C) and increased the ratio of Bax/Bcl-2 (6.75 ± 0.39, *p* < 0.01 compared with control; Figure 3D). However, pretreatment of Dap (10 μM) before NMDA exposure significantly reversed the levels of Bax (1.35 ± 0.24 fold of the control, *p* < 0.01 compared with NMDA alone; Figure 3B) and Bcl-2 (0.73 ± 0.13 fold of the control, *p* < 0.05 compared with NMDA alone; Figure 3C), and the ratio of Bax/Bcl-2 (2.86 ± 0.37, *p* < 0.01 compared with NMDA alone; Figure 3D).

### 2.4. Effects of Dap on Expression of NR2A- and NR2B-Containing NMDARs

**Figure 4:**
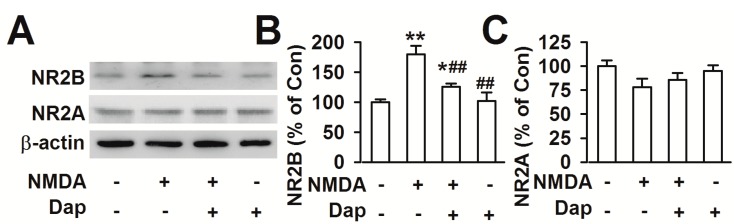
Effects of Dap on the NMDA receptors (**A**) Expression levels of NR2A and NR2B proteins were detected by western blot. (**B**) The result showed that the levels of NR2B markedly increased after exposure to NMDA in cultured cortical neurons. Pretreatment of 10 μM Dap significantly prevented the increase of NR2B by NMDA exposure. (**C**) Neither NMDA nor Dap treatment influenced the levels of NR2A. The data were pooled from five independent experiments. * *p* < 0.05, ** *p* < 0.01 compared with control, ^#^ *p* < 0.05, ^##^ *p* < 0.01 compared with NMDA alone.

Blockage of NR2B-containing NMDA receptors promote neuronal survival, exerting a protective action against NMDA receptor-mediated neuronal damage [11,12]. This is consistent to our results that NR2B specific antagonist Ro25-6981 abolished the NMDA-induced cell loss (Figure 1B). Western blot was performed to examine the effects of Dap on the expression of NMDAR subtypes. Levels of NR2B subtype expression was markedly increased after exposure to NMDA (180.0% ± 13.9% of the control; *p* < 0.01; Figure 4A,B). Pretreatment of Dap (10 μM) significantly reduced the over-expression of NR2B subtype induced by NMDA exposure (126.3% ± 5.4% of the control; *p* < 0.01 compared with NMDA alone; Figure 4A,B). However, neither NMDA exposure nor Dap (10 μM) treatment altered the levels of NR2A subtype receptors (Figure 4A,C). Pretreatment of Dap (10 μM) alone did not change the levels of NR2A and NR2B expression. Thus, downregulation of NR2B-containing NMDARs by Dap is suggested to be, at least partly, responsible for the neuroprotective effects against NMDA-induced excitotoxicity.

### 2.5. Inhibition of NMDA-Induced Ca^2+^ Overload by Dap

**Figure 5:**
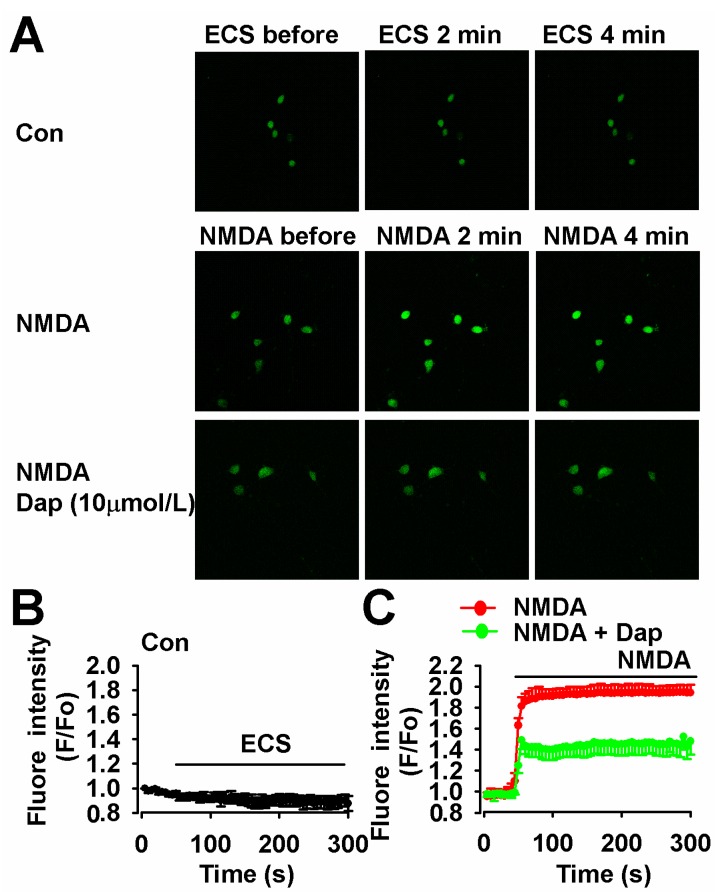
Effects of Dap on the calcium influx. (**A**) The green fluorescence under the laser scanning microscope at different time showed the concentration of calcium in neurons. (**B**) The fluorescence intensity showing the Ca^2+^ concentration was stable during detection by a laser scanning microscope for 300 s (n = 35 neurons). (**C**) 200 μM NMDA could evoke strong fluorescence intensity (n = 41 neurons) and pretreatment of Dap (10 μM) could significantly reduce fluorescence intensity in neurons (n = 36 neurons). Scale bar: 20 μm.

Sustained intracellular Ca^2+^ overload contributes to neuronal injury following excessive activation of NMDARs [13]. Next, we measured the calcium influx in cultured cortical neurons with treatment of NMDA or Dap. The fluorescence intensity can be regarded as an indicator of cytoplasmic Ca^2+^ concentration. The Ca^2+^ concentration in cultured neuron was stable during our experiments (Figure 5A,B). NMDA (200 μM) evoked a fast elevation of Ca^2+^ concentration for the next four minutes (Figure 5A,C). Pretreatment with Dap (10 μM, 24 h) could attenuate the amplitude of elevation in Ca^2+^ influx after NMDA exposure (Figure 5A,C).

### 2.6. Neuroprotection of Dap against Cerebral Ischemia-Reperfusion Insult

**Figure 6:**
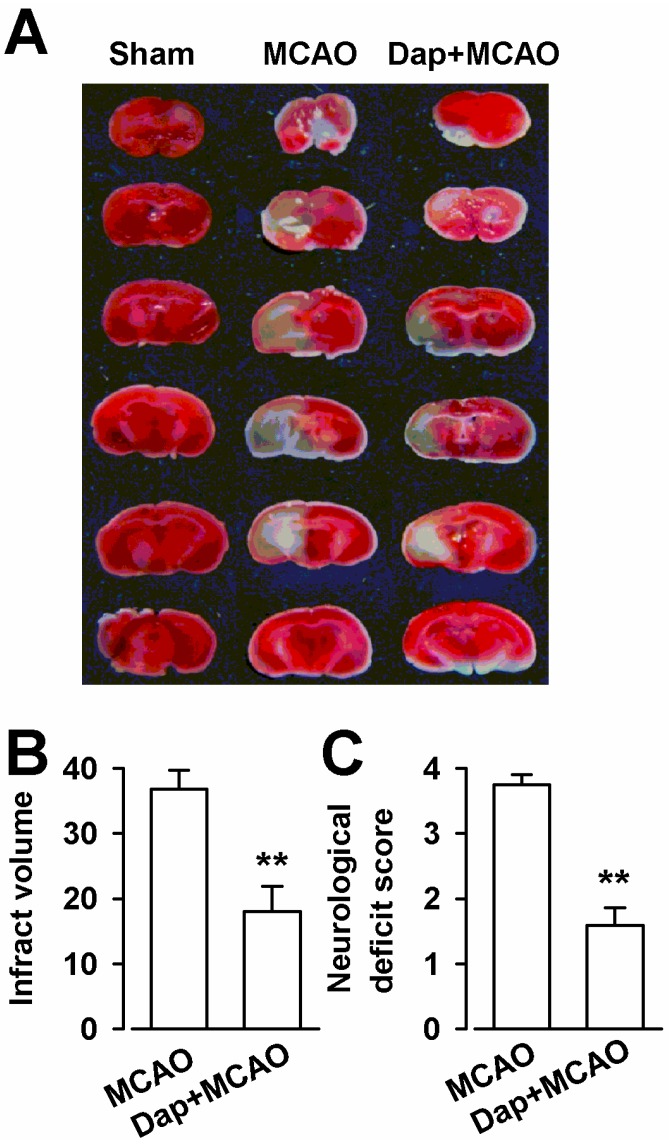
Effects of Dap on infarction volume and neurological deficit score after MCAO in mice. (**A**) Representative photograph of TTC staining of coronal brain sections (1 mm thick) from mice subjected to MCAO. Non-ischemic region is in red and the infarct region appears in white. (**B**) Infarct volume was measured at 24 h after MCAO. (**C**) The neurological deficit scores of mice were measured at 24 h after MCAO. n = 6, ** *p* < 0.01 compared with MCAO group.

The neuroprotective effect of Dap against ischemia-reperfusion injury by MCAO was evaluated via infarct volume, neurological deficit, and Nissl staining. As shown in Figure 6A,B, MCAO resulted in a large infarct volume (36.8% ± 2.9%) in the brain. Dap (100 mg/kg) significantly reduced the infarct volume (18.0% ± 3.8%, *p* < 0.01) compared with MCAO. Meanwhile, the results of the Zea Longa test showed that the neurological deficit score was significantly increased to 3.7 ± 0.1 in the MCAO group (*p* < 0.01) compared with the sham (Figure 6C). The neurological deficit was attenuated and the scores decreased to 1.6 ± 0.3 in the Dap + MCAO group (*p* < 0.01) compared with MCAO (Figure 6C).

Dap is one of secondary metabolites of plants used in folk medicine. This compound has showed neuroprotective activity and prevention of chronic unpredictable stress-induced cognitive deficits [3,14]. Glutamate plays a key role in neural transmission, development and synaptic plasticity. In the meantime, excessive accumulation of glutamate results in over activation of NMDA receptors, which induces excitotoxicity and causes neuronal damage [15]. Furthermore, activation of glutamate receptors triggers large increases in Ca^2+^ levels into neurons. Intracellular Ca^2+^ accumulation results in glutamate-induced neurotoxicity [16]. Ca^2+^ acts as an important second messenger, activating several intracellular signaling cascades including the PKC. It has been found that Dap shows inhibition of tyrosine phosphorylation and PKC activities [8]. In the present study, we cannot conclude that daphnetin directly targets the NR2B receptor. The accumulation of glutamate causes excitotoxicity by activating NMDA receptors and the Ca^2+^ accumulation in the cytoplasm. Dap shows inhibition of tyrosine phosphorylation and PKC activities [8], suggesting that Dap prevents the excitotoxicity through inhibiting the PKC activities by Ca^2+^ overload via the NMDA receptors.

Excitotoxicity has been linked to several pathological states of the nervous system such as seizures, ischemia, anoxia, inflammation, and neurodegenerative disorders [16]. In this study, NMDA exposure greatly decreased the cell viability in cultured cortical neurons. However, Dap treatment dose-dependently attenuated the cell loss. Hoechst 33258 and PI double staining further testified the neuroprotective effects of Dap, which was related to regulation of Bcl-2 and Bax expression. Western blot assays revealed the different roles of NR2A- and NR2B-containing NMDARs in the neuroprotection of Dap. Treatments of NMDA and Dap did not change the expression of NR2A receptors, but NMDA exposure increased the expression of NR2B, which is implied in mediating the excitotoxicity [11]. In the current study, a model of transient focal cerebral ischemia in mice induced by a 2 h MCAO and 24 h reperfusion was also used to estimate the neuroprotective effects of Dap. Dap showed infract volume reduction in the brain of mice, which received MCAO and improved the neurological deficit score. For cell experiment, Dap was administered for 24 h, and then NMDA was given in the same medium for additional 30 min. For animal experiment, Dap was administered after MCAO. It indicates the preventive and therapeutic activities of Dap in the treatment of excitotoxicity.

Several studies report the neuroprotective activity of coumarin derivatives in the culture cells or in the animal model of ischemic brain injury [9,17,18]. However, the protective mechanisms are mainly focused on the anti-oxidatant and anti-apoptotic activities through regulating the expression of apoptosis-related proteins [17], and maintaining the cellular levels of glutathione and superoxide dismutase activity [9]. Our present study showed that treatment of Dap selectively reversed the protein levels of NR2B induced by NMDA exposure, implying that the neuroprotection of Dap is likely to antagonize a particular NMDAR subunit. However, the conclusion that neuroprotection of Dap is partially through downregulation of NR2B-containing NMDA receptors is from the neuronal cultures studies* in vitro*. Further studies will be conducted to elucidate the mechanisms* in vivo*. This phenomenon is quite beneficial for neurodegenerative diseases relevant to glutamate excitotoxicity. Our present study sheds a light on the mechanisms underlying neuroprotection of natural product in addition to the anti-oxidant and anti-apoptotic activities.

## 3. Experimental

### 3.1. General Information

Dap was purchased from the ShangHai PureOne Biotechnology (Shanghai, China). Purity: 98% by High-performance liquid chromatography (HPLC). MTT [3-(4,5-dimethylthiazol-2-yl)-2,5-diphenyl- tetrazolium bromide], Ro25-6981 and anti-β-actin antibody were purchased from Sigma (St. Louis, MO, USA). Anti-NR2A was purchased from Millipore (Billerica, MA, USA). Anti-NR2B, anti-Bax, and anti-Bcl-2 antibodies were purchased from Chemicon (Temecula, CA, USA). Dulbecco’s Modified Eagle’s Medium (DMEM) was purchased from Hyclone (Logan, UT, USA). Fetal bovine serum, Neurobasal medium, and B27 were got from Gibco (Invitrogen, Carlsbad, CA, USA). All of the other chemicals and reagents were standard commercially available biochemical quality.

### 3.2. Primary Mouse Cortical Neuronal Culture

The Animal Care and Use Committee of the Fourth Military Medical University approved all animal protocols. Cultured prefrontal cortex neurons were derived from E18 C57Bl/6 mice as Wang* et al.* described [19]. Briefly, the prefrontal cortex was dissected, minced, and trypsinized for 15 min using 0.125% trypsin (Invitrogen). Cells were then seeded onto 96-well plates, 24-well plates, 6-well plates, or 100 mm dishes. All plates were pre-coated with 50 μg/mL poly-d-lysine (Sigma) and grown in Neurobasal medium (Invitrogen) supplemented with B27 and 2 mM glutamine (Invitrogen). In B27/Neurobasal medium, glial growth was reduced to less than 0.5% as assessed by immunocytochemistry for glial fibrillary acidic protein (GFAP). The vast majority of cultured cells were immunoreactive for neuron-specific enolase [20]. The cultures were incubated at 37 °C in 95% air/5% carbon dioxide with 95% humidity. Cultures were used for experiments on the 10th day* in vitro* (DIV 10). The neurons were briefly rinsed with phosphate-buffered saline (PBS) and added new Neurobasal medium without B27, and then treated with Dap for 24 h. Then, NMDA and glycine (10 μM) were added to the medium with Dap for another 30 min. Glycine is a co-activator of the NMDA receptors and was used with NMDA to induce NMDA-mediated toxicity. The cells were washed twice and returned to the original culture medium for another 24 h.

### 3.3. Cell Viability Analysis

The MTT assay was used to detect cell viability as described by Liu* et al.* [21]. Neurons were cultured in 96-well plates at a density of 1 × 10^4^ per well. The substrate MTT was dissolved in DMEM medium and added to each well at a final concentration of 0.5 mg/mL and then incubated at 37 °C for 4 h. Then the medium was then replaced by 150 μL dimethyl sulfoxide (DMSO) to dissolve the formazan product. The optical density (OD) was read on a Universal Microplate Reader (Elx 800, Bio-TEK instruments Inc., Winooski, VT, USA) at 570 nm (using 630 nm as a reference). Cell viability was presented as a percentage of the absorbance of untreated cultures. All data are expressed as mean ± SEM of three independent experiments and each mean included data from six wells.

### 3.4. Hoechst 33258 and PI Double Staining

Cell death was determined by propidium iodide (PI, Sigma) and Hoechst 33258 (Sigma) double fluorescent staining as previously described [22]. Neurons were cultured in 24-well plates at a density of 600 cells/mm^2^. Twenty-four hours after NMDA treatment, the cells were stained with PI (10 μg/mL) and Hoechst 33258 (10 μg/mL) for 15 min, and then fixed in 4% paraformaldehyde for 10 min. Hoechst 33258 is excited by UV light at around 350 nm and emits blue fluorescence light at 461 nm. Hoechst 33258 is often used to distinguish the compact chromatin of apoptotic nuclei from that of normal cells. Propidium iodide, a red-fluorescence dye (excited at 620 nm), is only permeant to dead cells. Staining was imaged and analyzed using a Fluoview FV100 instrument (Olympus, Tokyo, Japan). To assess apoptotic nuclei and dead/dying neurons, three visual fields were randomly selected from each well.

### 3.5. Western Blot Analysis

In order to further explore the mechanisms involved in DAP-mediated neuroprotection, we examined the effects of DAP on signaling pathways related to survival by western blot analysis as described previously [23]. Neurons were cultured in 6-well plates at a density of 2 × 10^6^ cells/well. After each treatment, cells were rinsed twice with PBS and lysed by M-PER Protein Extraction Buffer containing 1× protease inhibitor cocktail. Cell protein was quantified by a BCA Kit and equal amounts of protein (50 μg) separated on 10% polyacrylamide gel followed by transferred onto an Immun-Blot PVDF membrane. The membrane was blocked for 1 h with 5% non-fat milk in Tris-Phosphate buffer containing 0.05% Tween 20 (TBS·T). It was further incubated overnight at 4 °C with primary antibodies including anti-NR2A (dilution ratio 1:400), anti-NR2B (dilution ratio 1:1000), anti-Bax (dilution ratio 1:400), anti-Bcl-2(dilution ratio 1:400), and β-actin (dilution ratio 1:10000) as a loading control. The membranes were incubated with horseradish peroxidase conjugated secondary antibodies (anti-rabbit IgG for the primary antibodies), and bands were visualized using an ECL system (Perkin Elmer, Zaventem, Belgium).

### 3.6. Calcium Imaging

Calcium imaging was performed as previously described [24]. Neurons were cultured in 3.5 mm plates made especially for laser scanning microscope at a density 3 × 10^5^ per plate. Cultured cells were washed twice using Mg^2+^-free extracellular solution (ECS) containing (in mM): NaCl, 140; KCl, 3; CaCl_2_, 2; HEPES, 10; and glucose, 10. The pH was adjusted to 7.2 to 7.3 with NaOH and osmotic pressure adjusted to 310 ± 5 with sucrose. Then, the neurons were incubated with 2 μM fluo-3/AM at 37 °C. After 30 min, the cultures were washed twice and returned to the original culture medium for an additional 30 min. The dye-loaded cells were measured for fluorescence using a confocal laser scanning microscope (FV1000, Olympus). Prior to NMDA application, the dye-loaded cells were scanned for approximately 1 min to obtain a basal level of intracellular Ca^2+^. Then, 200 μM NMDA was applied to the cultures, and an equal amount of ECS was added as a placebo. Dap was added 24 h before the experiments and present in the whole experimental process. The change of Ca^2+^ concentration was estimated by the fluorescence ratio of the fluo-3/AM-loaded neurons for another 4 min. The results are expressed as changes relative to basal levels, and five cells were selected randomly for analysis.

### 3.7. Middle Cerebral Artery Occlusion (MCAO)

The experimental protocol used in the present study was approved by the Animal Care and Use Committee of the Fourth Military Medical University. C57 mice (25–30 g) were provided by the Experimental Animal Center of the Fourth Military Medical University. The male mice were housed with food and water available *ad libitum* in a colony room at controlled temperature (24 ± 2 °C), humidity (50%–60%), and 12:12 h light–dark cycle. MCAO was performed according to previously described methods with some modifications [25]. In brief, mice were anesthetized with chloral hydrate (300 mg/kg) and a longitudinal incision of 10 ± 2 mm was made along the midline of the ventral cervical part. The right common carotid artery, internal carotid artery (ICA), and external carotid artery (ECA) were exposed and carefully isolated. Anylon monofilament (20 mm length and 0.2 mm diameter) was inserted from the lumen of the ECA to that of the right ICA to occlude the origin of the right middle cerebral artery (MCA). The right MCA was occluded for 120 min, and thereafter the brain was allowed to be reperfused with blood by withdrawing the nylon monofilament. Temperature was maintained at 37 ± 0.5 °C throughout the surgery. Animals were randomly assigned to three groups: sham, MCAO, Dap + MCAO. The mice in sham were subjected to surgery and exposed the right ICA and the right ECA but did not suffer MCAO. The MCAO mice were administered the vehicle or drugs (physiological saline or Dap, 100 mg/kg respectively) by peritoneal injection immediately after the surgery.

### 3.8. Neurobehavioral Evaluation

Neurobehavioral evaluation and infarct volume assessment were performed in each group (n = 6 for each group). The mice were neurologically assessed by an investigator who was unaware of the animal grouping 24 h after MCAO. Neurological deficit was determined as described [26]. Neurological evaluation was scored using a 5-point scale: 0, no neurological deficit; 1, failure to extend left forepaw fully; 2, circling to the left; 3, inability to bear weight on the left; 4, no spontaneous walking with depressed level of consciousness.

### 3.9. Infarct Volume Assessment

After neurological evaluation, the mice were re-anesthetized with an overdose of pentobarbital sodium, and then decapitated. The brain was rapidly removed and cooled in iced saline for 10 min. Coronal sections (1 mm) were cut and immersed in 2% TTC at 37 °C for 30 min and then transferred to 4% buffered formalin solution for 24 h fixation. The brain slices were photographed using a digital camera. Unstained areas were defined as infarct and were measured using image analysis software (Adobe Photoshop CS3 for Windows). The infarct volume was calculated by measuring the unstained area in each slice, multiplying it by slice thickness (1 mm), and then summing all five slices.

### 3.10. Data Analysis

Data were expressed as the mean ± SEM. Statistical comparisons were evaluated by a *t-*test and one-way ANOVA was used for comparison among multiple groups. *p* < 0.05 was considered to be statistically significant.

## 4. Conclusions

In summary, the presented results suggest that the neuroprotection of Dap is, at least partially, associated with the down-regulation of NR2B-containing NMDARs and the Ca^2+^ accumulation. This neuroprotective effect is closely related to the inhibition of apoptosis and Ca^2+^ overload induced by NR2B-containing NMDARs. The present study may shed light upon the pharmacological basis for clinical application of Dap.
